# Complement and IgM in FSGS: Minor Characters or Leading Actors?

**DOI:** 10.34067/KID.0000000814

**Published:** 2025-08-28

**Authors:** Andrea Angeletti, Paolo Cravedi

**Affiliations:** 1 Nephrology, Dialysis and Transplantation Unit, IRCCS Istituto Giannina Gaslini, Genoa, Italy; 2 Department of Medicine, Translational Transplant Research Center, Icahn School of Medicine at Mount Sinai, New York, New York

**Keywords:** complement, biomarkers

FSGS is a histopathologic pattern of glomerular injury resulting from diverse clinicopathologic conditions, all of which converge on podocyte injury as the primary pathogenic mechanism, ultimately leading to segmental glomerular sclerosis. The prevalence of FSGS has been rising in recent decades, making it the most common glomerular cause of ESKD. Primary FSGS, which typically presents with nephrotic syndrome, is believed to be driven by circulating permeability factors that induce podocyte foot process effacement. Secondary FSGS encompasses maladaptive forms due to glomerular hyperfiltration—such as in obesity or nephron mass reduction—as well as virus-associated and drug-induced variants that result in direct podocyte injury. In addition, genetic abnormalities are increasingly recognized as causative factors in certain FSGS subtypes.^1^ Notably, glomerulosclerosis may continue to progress even after the underlying disease enters remission, suggesting the existence of common downstream pathways driving glomerular disease progression. Understanding these mechanisms could provide novel therapeutic targets to prevent long-term kidney function decline in FSGS and other glomerular disorders.

IgM and C3 deposits are often seen in the glomeruli of patients with FSGS and may have functional implications. IgM and C3 deposits in the kidney are associated with worse clinical outcomes.^2^ In addition, the presence of complement activation products in urine and plasma has been linked to worse disease severity and impaired therapeutic responses,^3^ indicating that complement activation may serve as a surrogate biomarker for disease progression.

In the present issue, Caliskan *et al.*^4^ investigated the association of glomerular IgM and C3 immunostaining with FSGS activity and clinical outcomes in the Cure Glomerulopathy Network FSGS cohort. Cure Glomerulopathy Network is a multicenter prospective observation cohort study of children and adults with biopsy-proven glomerular diseases and the present study cohort included 175 FSGS patients. Glomerular IgM, C3, and IgG deposits were observed in 88 (50%), 48 (27.4%), and 27 (15.4%) patients, respectively, indicating that these findings are common. C3 deposition was correlated with more severe histologic lesions, including global sclerosis, tubular microcystic changes, interstitial inflammation, interstitial fibrosis/tubular atrophy, and tip lesions.

The authors also measured the urinary levels of membrane attack complex (MAC), the terminal complement activation product, which correlated with total segmental sclerosis, interstitial fibrosis/tubular atrophy, and interstitial inflammation in renal biopsies. Only urinary MAC and age at enrollment were significantly associated with the composite outcome of ESKD or a 40% eGFR decline in the adjusted Cox survival models.

Previous studies had identified an association between complement split products in the urine and proteinuria in FSGS and other glomerular diseases. Although the association may simply be due to the loss of selectivity in the glomerular filtration barrier leading to protein loss in the urine (including complement components), the statistical adjustments in this study support an independent relationship between urinary MAC and ESKD.^5^

In the study by Caliskan *et al.*,^4^ the prevalence of concomitant IgM/C3 deposition was similar in patients with and without active disease, suggesting no synergistic effect between IgM and C3. However, the frequent detection of IgM and C3 in this and other reports suggests that, in FSGS glomeruli, complement is activated by IgM through the classical pathway.

Peng *et al.*^6^ found that glomerular capillary C3 deposition is a significant risk factor for unfavorable kidney outcomes in pediatric patients with primary FSGS, particularly when associated with IgM deposition. Mirioglu *et al.*^7^ emphasized that the codeposition of IgM and C3 serves as a predictor of unfavorable kidney outcomes in adult patients with primary FSGS.

On injury, glomerular endothelial cells express new autoantigens that are targeted by natural IgM. Although the identity of the glomerular epitopes bound by IgM in FSGS is not entirely known, Thurman's groups showed that IgM in FSGS patients binds to cardiolipin or other phospholipid epitopes on the surface of injured endothelial cells. Consistently, anticardiolipin IgM increases in mice with adriamycin-induced FSGS and patients with nephrotic syndrome have higher levels of anticardiolipin IgM than healthy or disease controls.^8^

Additional evidence supporting a pathogenic role of IgM in FSGS pathophysiology comes from recent studies demonstrating that combined B-cell and plasma-cell depletion with anti-CD20 and anti-CD38 antibodies effectively induced disease remission in multidrug-resistant FSGS and recurrent post-transplant FSGS.^9^ Notably, patients with IgM levels below the median at 3 months post-treatment exhibited a significantly lower relapse rate at 9 months than those with higher IgM levels, suggesting a mechanistic link between IgM and disease activity. Distinguishing the role of low-affinity natural IgM versus T-cell–dependent high-affinity IgM clones may be critical to elucidate FSGS pathogenesis and mechanisms of glomerular disease progression.

Prior data also suggest that complement can activate in the glomeruli of FSGS patients through the alternative pathway as a consequence of the cleavage of the key complement regulator decay accelerating factor (CD55) on the membranes of injured podocytes.^10^ Therefore, it is possible that different mechanisms (IgM-mediated and IgM-independent) activate complement in FSGS, possibly in different phases of the disease.

Once activated, the complement cascade promotes glomerular injury through multiple effector mechanisms. We have shown that activation of the C3a receptor promotes podocyte cytoskeleton disruption through an IL-1b–mediated mechanism. Based on this background, we treated a series of FSGS patients with the anti–IL-1Rb antibody Anakinra with encouraging results.^11^

C3 deposition in kidney tissues has been associated with the progression of kidney fibrosis through several other mechanisms. Studies have shown that C3 promotes renal fibrosis by inducing IL-17A secretion, facilitating M1 macrophage polarization, and activating toll-like receptor 4/NFκB signaling, contributing to podocyte injury and fibrosis.^4^ Animal data also suggest that preventing complement activation by increasing the expression of complement regulator decay accelerating factor or by blocking C3a receptor has beneficial effects on proteinuria and glomerulosclerosis.

Collectively, these experimental and clinical findings highlight the active involvement of IgM and the complement system in the pathophysiology of FSGS. This evidence provides a foundation for future research aimed at identifying novel biomarkers and therapeutic targets. Furthermore, integrating transcriptomic and proteomic data from FSGS-affected glomeruli may clarify whether complement activation and IgM deposition contribute directly to podocyte injury or represent secondary effects of glomerular damage. Such insights could refine disease classification and inform targeted therapeutic strategies.

Urinary MAC may serve as a biomarker for identifying patients at the highest risk of progression to ESKD. If validated and standardized, it could function as a risk stratifier in clinical trials, aid in personalized treatment strategies, and potentially facilitate therapy monitoring. Given that MAC represents the terminal pathway of complement activation, assessing additional complement split products may be critical for tailoring anti-complement therapies based on the predominant pathway involved in individual patients.

Owing to the pathogenic role of complement in FSGS, it is also tempting to speculate that targeting complement cascade represents a valuable strategy to retard or prevent FSGS progression, especially considering the excellent safety profile of anti-complement therapies. A completed clinical trial (NCT05314231), whose results have not been released yet, tested the effect of a C5 inhibitor in multiple proteinuric glomerular diseases, including FSGS. These results will provide important information on the utility of blocking complement in treating FSGS.

The findings by Caliskan *et al.*,^4^ together with prior evidence, underscore the potential of urinary complement split products as biomarkers for risk stratification and treatment monitoring in FSGS. Identifying specific complement split products may help determine the predominant pathway involved in individual patients, enabling a more personalized approach to complement-targeted therapies. To validate this hypothesis, routine evaluation of glomerular IgM and complement deposition, along with urinary complement split products, should be integrated into renal clinical trials.
